# Effects of Dapagliflozin in Chronic Kidney Disease Across the Spectrum of Age and by Sex

**DOI:** 10.1007/s11606-023-08397-9

**Published:** 2023-12-14

**Authors:** Margaret K. Yu, Priya Vart, Niels Jongs, Ricardo Correa-Rotter, Peter Rossing, John J.V. McMurray, Fan-Fan Hou, Walter Douthat, Dinesh Khullar, Anna Maria Langkilde, David C. Wheeler, Hiddo J. L. Heerspink, Glenn M. Chertow

**Affiliations:** 1 Department of Medicine, Stanford University School of Medicine, Stanford, CA USA; 2 Department of Clinical Pharmacy and Pharmacology, University of Groningen, University Medical Centre Groningen, Groningen, The Netherlands; 3 Department of Internal Medicine, University of Groningen, University Medical Center Groningen, Groningen, The Netherlands; 4 The National Medical Science and Nutrition Institute Salvador Zubiran, Mexico City, Mexico; 5 Steno Diabetes Center Copenhagen, Gentofte, Denmark; 6 Department of Clinical Medicine, University of Copenhagen, Copenhagen, Denmark; 7 Institute of Cardiovascular and Medical Sciences, University of Glasgow, Glasgow, UK; 8 Division of Nephrology, Nanfang Hospital, Southern Medical University, National Clinical Research Center for Kidney Disease, Guangzhou, China; 9 Department of Nephrology, Hospital Privado Universitario de Cordoba, Cordoba, Argentina; 10 Department of Nephrology and Renal Transplant Medicine, Max Super Speciality Hospital, Saket, New Delhi, India; 11 BioPharmaceuticals R&D, AstraZeneca, Gothenburg, Sweden; 12 Department of Renal Medicine, University College London, London, UK; 13 The George Institute for Global Health, Sydney, New South Wales Australia; 14 Department of Epidemiology and Population Health, Stanford University School of Medicine, Stanford, CA USA; 15 Department of Medicine, Stanford University School of Medicine, Stanford, CA USA

**Keywords:** SGLT2 inhibitor, CKD, age differences, sex differences

## Abstract

**Background:**

The sodium-glucose cotransporter type 2 inhibitor dapagliflozin reduces the risk of progressive kidney disease and cardiovascular events in patients with chronic kidney disease, with and without type 2 diabetes. Whether its effects are uniform across the spectrum of age and among men and women is unknown.

**Objective:**

We performed a pre-specified analysis in DAPA-CKD to evaluate efficacy and safety of dapagliflozin according to baseline age and sex.

**Design:**

Prospective randomized placebo-controlled trial.

**Participants:**

A total of 4304 adults with chronic kidney disease (estimated glomerular filtration rate (eGFR) 25–75 mL/min/1.73 m^2^; urinary albumin-to-creatinine ratio 200–5000 mg/g) with and without type 2 diabetes.

**Intervention:**

Dapagliflozin 10 mg versus placebo once daily.

**Main Measures:**

Primary endpoint was a composite of ≥ 50% sustained eGFR decline, end-stage kidney disease, and kidney or cardiovascular death. Secondary endpoints included kidney composite endpoint (same as primary composite endpoint but without cardiovascular death), cardiovascular composite endpoint (hospitalized heart failure or cardiovascular death), and all-cause mortality.

**Key Results:**

Median follow-up was 2.4 years. Absolute risks of cardiovascular composite endpoint and all-cause mortality were higher in older patients. Absolute risk of kidney composite endpoint was highest in patients < 50 years (10.7 and 6.2 per 100 patient-years in the placebo and dapagliflozin groups, respectively) and lowest in patients ≥ 80 years (3.0 and 1.2 per 100 patient-years in the placebo and dapagliflozin groups, respectively). There was no evidence of heterogeneity of the effects of dapagliflozin on the primary or secondary endpoints based on age or sex. Neither age nor sex modified the effects of dapagliflozin on total or chronic eGFR slope.

**Conclusions:**

Dapagliflozin reduced the risks of mortality, cardiovascular events, and CKD progression in older patients, including in septuagenarians and octogenarians who comprised 25% of participants. Ageism and/or therapeutic nihilism should not discourage the use of dapagliflozin in older women and men who are likely to experience considerable benefit.

**Trial Registry:**

clinicaltrials.gov

**NIH Trial Registry Number:**

NCT03036150

**Supplementary Information:**

The online version contains supplementary material available at 10.1007/s11606-023-08397-9.

## INTRODUCTION

Sodium-glucose cotransporter type 2 (SGLT2) inhibitors reduce the risk of progressive kidney disease and cardiovascular events in patients with chronic kidney disease (CKD). In the DAPA-CKD trial,^1^ dapagliflozin reduced the risks of progressive kidney disease, hospitalized heart failure or cardiovascular death, and all-cause mortality in patients with CKD and severe albuminuria with and without type 2 diabetes. Similar risk reductions were reported among patients with CKD and type 2 diabetes in CREDENCE^2^ and among patients with CKD with and without diabetes in EMPA-KIDNEY.^3^

Despite these advances, persons with CKD still experience distressingly high rates of mortality and morbidity relative to persons without CKD and these risks vary based on age and sex.^4–5^ *Absolute* risks of cardiovascular events (including cardiovascular mortality) and kidney failure are higher in older compared with younger patients with CKD,^4–7^ whereas *relative* risks of death,^6^ cardiovascular disease,^7^ and rates of CKD progression^8^ are higher in younger patients. Whether the benefits of dapagliflozin are consistent across the spectrum of age, or among women and men, is unknown.

With respect to sex differences in CKD, women with CKD have a lower risk of CKD progression,^9^ cardiovascular events,^10^ and mortality compared with men.^10^ Given sex differences in the efficacy and safety of several commonly prescribed cardiovascular medications, including angiotensin-converting enzyme (ACE) inhibitors and statins,^11^ it is essential to understand whether sex modifies the effects of dapagliflozin in CKD.

## METHODS

DAPA-CKD was a randomized, double-blind, placebo-controlled, multicenter clinical trial; manuscripts describing trial design, baseline characteristics, primary results, and results stratified by diabetes status, history of cardiovascular disease, and several other baseline clinical characteristics have been previously published.^1, 12^ The trial was sponsored by AstraZeneca and conducted at 386 sites in 21 countries from February 2017 through June 2020 and registered at clinicaltrials.gov (NCT03036150). All participants provided written informed consent before any study-specific procedure commenced. The safety of participants in the trial was overseen by an independent Data and Safety Monitoring Committee. The trial was conducted according to the principles of the Declaration of Helsinki. Ethics committees at all participating centers approved the protocol, and all participants provided informed consent.

### Participants

Adults with or without type 2 diabetes, and with an estimated glomerular filtration rate (eGFR) 25–75 mL/min/1.73 m^2^ and urinary albumin-to-creatinine ratio (UACR) 200–5000 mg/g, were eligible for participation. We required patients to be treated with a stable maximally tolerated dose of renin-angiotensin-aldosterone system (RAAS) inhibitor (angiotensin-converting enzyme [ACE] inhibitor or angiotensin receptor blocker [ARB]) for ≥ 4 weeks, unless medically contraindicated. Key exclusion criteria included documented diagnosis of type 1 diabetes, polycystic kidney disease, lupus nephritis, or anti-neutrophil cytoplasmic antibody-associated vasculitis. A complete list of inclusion and exclusion criteria and the trial protocol have been previously published.^1, 12^

### Procedures

Participants were randomly assigned to dapagliflozin 10 mg once daily or matching placebo, in accordance with the sequestered, fixed-randomization schedule, with the use of balanced blocks to ensure an approximate 1:1 ratio of the two regimens. Randomization was stratified by diabetes status and UACR (≤ or > 1000 mg/g). We calculated eGFR using the Chronic Kidney Disease Epidemiology Collaboration (CKD-EPI) and incorporated results from the equation as originally defined,^13^ including a term for self-reported race (Black versus non-Black). Recruitment of patients with eGFR 60–75 mL/min/1.73 m^2^ was limited to no more than 10% of trial participants. Participants and all study personnel (except the Independent Data Monitoring Committee) were masked to treatment allocation. After randomization, in-person study visits were performed after 2 weeks and 2, 4, and 8 months and at 4-month intervals thereafter. At each follow-up visit, study personnel recorded vital signs, obtained blood and urine samples, and recorded information on potential study endpoints, adverse events, concomitant therapies, and study drug adherence.

### Endpoints

The primary composite endpoint was time to ≥ 50% decline in eGFR (confirmed by a second serum creatinine measurement after at least 28 days), onset of end-stage kidney disease (EKSD; defined as maintenance dialysis for at least 28 days, kidney transplantation, or eGFR < 15 mL/min/1.73 m^2^ confirmed by a second measurement after at least 28 days), or death from a kidney or cardiovascular cause. Secondary endpoints were time to (1) a composite kidney endpoint of ≥ 50% sustained decline in eGFR, EKSD, or death from kidney disease; (2) a composite cardiovascular endpoint defined as hospitalization for heart failure or cardiovascular death; and (3) death from any cause (all-cause mortality). We also assessed prespecified change in eGFR slope as an exploratory efficacy endpoint. All efficacy endpoints were adjudicated by a masked, independent Clinical Events Committee, except for the quantitative assessments of eGFR, which were obtained from our central laboratory.

### Categories of Age and Sex

Adults (18 years and above) were eligible to participate in DAPA-CKD. For the purpose of graphical representation, we categorized age by decade (< 50, 50–59, 60–69, 70–79, and ≥ 80 years) and sex, which was self-reported.

### Statistical Analysis

The overall analytic approach, power calculation, and pre-specified statistical analysis plan have been previously published.^1^ All analyses presented here followed the intention-to-treat principle. Briefly, we conducted time-to-event analyses using a proportional hazards (Cox) regression stratified by randomization factors (presence of type 2 diabetes and UACR ≤ 1000 or > 1000), adjusting for baseline eGFR, yielding hazard ratios (HR) and 95% confidence intervals (95% CI) from model parameter coefficients and standard errors, respectively. For the purpose of the current analysis, we evaluated the primary and secondary efficacy endpoints in participants stratified by baseline age category and sex. We tested for heterogeneity of the dapagliflozin treatment effect by including a multiplicative interaction term between randomized treatment group and age and sex. We further examined 3-way interactions by treatment assignment, age or sex, and diabetes status. For time to event analyses, we assessed for non-uniformity of HRs with the Akaike’s information criterion.

We analyzed the effects of dapagliflozin on the mean on-treatment eGFR slope by fitting a two-slope mixed-effects linear spline model (with a knot at week 2) with a random intercept and random slopes for treatment.^14^ The model included fixed effects for treatment, age or sex, stratification factors (diabetes status and UACR), and a continuous, fixed covariate for time to visit. To determine eGFR slopes for age or sex, we added to the model all possible interaction terms for treatment effect, age or sex, and time to visit, assuming an unstructured variance-covariance matrix. We computed the mean total slope as a weighted combination of the acute and chronic slopes to reflect the mean rate of eGFR change to until the last on-treatment visit. We also presented the pattern of change in mean eGFR using a restricted maximum-likelihood repeated-measures approach. This latter analysis included fixed effects of treatment, visit, treatment-by-visit interaction, and treatment-by-age or treatment-by-sex interaction. We added interaction terms between age or sex, visit, and treatment assignment to assess the change in eGFR within age or sex subgroups.

We considered 2-tailed *p* values < 0.05 to be statistically significant, without adjustment for multiple comparisons. We performed all analyses with SAS version 9.4 (SAS Institute) or R version 4.0.2 (R Foundation).

### Role of the Sponsor

AstraZeneca provided support for the conduct of the DAPA-CKD trial; AstraZeneca also provided support to InScience Communications for help in the preparation of figures. The analysis plan was conceived by independent members of the DAPA-CKD Steering Committee. Statistical analyses were conducted by Drs. Vart and Jongs at the University of Groningen. Drs. Yu and Chertow completed the first draft of the manuscript; co-authors edited sections of the manuscript, and all authors approved the final version for submission.

## RESULTS

### Baseline Characteristics

Study participants were followed for a median of 2.4 years. Baseline demographic and clinical characteristics, stratified by age (in decades) and sex, are shown in Tables 1 and 2, respectively. The age of the trial participants ranged from 18 to 93 years. Of the 4304 study participants, 671 (15.6%), 935 (21.7%), 1501 (34.9%), 999 (23.2%), and 198 (4.6%) participants were < 50, 50–59, 60–69, 70–79, and ≥ 80 years of age, respectively; 1425 (33.1%) were women, and 2879 (66.9%) were men. Racial composition varied by age and sex; older patients were more likely to be White, younger patients were more likely to be Asian, and a higher proportion of women were Black. More than two-thirds of participants (2906, 67.5%) had type 2 diabetes; the proportion of patients with diabetes was lowest in the youngest age group (33.1%). Older participants were less likely to be current smokers, had higher average blood pressure, and were more likely to have comorbid cardiovascular disease than younger participants. Women had a lower burden of cardiovascular disease than men. Within the range of trial inclusion criteria, mean eGFR and median UACR were higher in younger relative to older participants.

**Table 1:** Baseline Characteristics of the Study Population by Age Categories

Characteristic	Age < 50 years (*n* = 671)	Age 50–59 years (*n* = 935)	Age 60–69 years (*n* = 1501)	Age 70–79 years (*n* = 999)	Age ≥ 80 years (*n* = 198)
Age (years), mean (SD)	41.0 (6.9)	55.2 (2.8)	64.7 (2.9)	73.7 (2.8)	82.5 (2.6)
Sex (female), *n* (%)	223 (33.2)	296 (31.7)	520 (34.6)	316 (31.6)	70 (35.3)
Race/ethnicity, *n* (%)					
White	293 (43.7)	426 (45.6)	844 (56.2)	606 (60.7)	121 (61.1)
Black or African American	24 (3.6)	45 (4.8)	74 (4.9)	43 (4.3)	5 (2.5)
Asian	325 (48.4)	375 (40.1)	446 (29.7)	265 (26.5)	56 (28.3)
Others	29 (4.3)	89 (9.5)	137 (9.1)	85 (8.5)	16 (8.1)
Geographic region, *n* (%)					
Asia	304 (45.3)	348 (37.2)	409 (27.2)	236 (23.6)	49 (24.7)
Europe	193 (28.8)	248 (26.5)	446 (29.7)	298 (29.8)	48 (24.2)
North America	76 (11.3)	140 (15.0)	291 (19.4)	240 (24.0)	66 (33.3)
Latin/South America	98 (14.6)	199 (21.3)	355 (23.6)	225 (22.5)	35 (17.7)
Current smoker, *n* (%)	102 (15.2)	166 (17.7)	210 (14.0)	96 (9.6)	10 (5.0)
SBP (mmHg), mean (SD)	130.2 (16.4)	135.2 (16.5)	138.6 (17.1)	140.3 (17.8)	141.3 (17.0)
DBP (mmHg), mean (SD)	81.3 (11.1)	79.9 (9.3)	77.4 (10.2)	74.1 (9.8)	71.8 (10.6)
BMI (kg/m^2^), mean (SD)	27.9 (6.5)	29.5 (6.4)	30.4 (6.1)	29.5 (5.8)	28.4 (4.9)
HbA1c (%), mean (SD)	6.2 (1.7)	7.2 (1.9)	7.3 (1.7)	7.1 (1.5)	6.9 (1.3)
eGFR (mL/min/1.73 m^2^), mean (SD)	43.3 (12.6)	43.9 (12.9)	43.4 (12.4)	42.2 (11.7)	40.0 (10.7)
UACR (mg/g), median (IQR)	1052 (564, 1981)	1037 (514, 2280)	971 (478, 1824)	824 (414, 1704)	750 (417, 1413)
Diabetes (yes), *n* (%)	222 (33.1)	619 (66.2)	1151 (76.7)	777 (77.8)	137 (69.2)
Diabetes duration (years), median (IQR)	8.4 (4.4, 14.6)	11.6 (5.9, 18.8)	14.5 (7.8. 21.0)	16.2 (9.9, 22.8)	17.6 (8.5, 28.0)
CV disease (yes), *n* (%)	84 (12.5)	267 (28.6)	635 (42.3)	518 (51.8)	106 (53.5)
ACE inhibitor/ARB (yes), *n* (%)	649 (96.7)	911 (97.4)	1455 (96.9)	969 (97.0)	190 (96.0)
Diuretics (yes), *n* (%)	200 (29.8)	379 (40.5)	706 (47.0)	503 (50.3)	94 (47.5)
Insulin (yes), *n* (%)*	115 (51.8)	343 (55.4)	678 (58.9)	408 (52.5)	54 (39.4)

*ACE* angiotensin-converting enzyme, *ARB* angiotensin receptor blocker, *BMI* body mass index, *CV* cardiovascular, *DBP* diastolic blood pressure, *eGFR* estimated glomerular filtration, *SBP* systolic blood pressure, *UACR* urinary albumin-creatinine ratio

*In those with diabetes

**Table 2:** Baseline Characteristics of the Study Population by Sex

Characteristic	Female (*n* = 1425)	Male (*n* = 2879)
Age (years), mean (SD)	61.9 (11.9)	61.8 (12.2)
Race/ethnicity, *n* (%)		
White	694 (48.7)	1596 (55.4)
Black or African American	86 (6.0)	105 (3.6)
Asian	492 (34.5)	975 (33.9)
Others	153 (10.7)	203 (7.0)
Geographic region, *n* (%)		
Asia	462 (32.4)	884 (30.7)
Europe	384 (26.9)	849 (29.5)
North America	252 (17.7)	561 (19.5)
Latin/South America	327 (22.9)	585 (20.3)
Current smoker, *n* (%)	89 (6.2)	495 (17.2)
SBP (mmHg), mean (SD)	136.9 (18.3)	137.2 (17.0)
DBP (mmHg), mean (SD)	77.1 (10.1)	77.7 (10.6)
BMI (kg/m^2^), mean (SD)	29.8 (6.9)	29.4 (5.8)
HbA1c (%), mean (SD)	7.3 (1.9)	7.0 (1.6)
eGFR (mL/min/1.73 m^2^), mean (SD)	42.5 (12.4)	43.4 (12.3)
UACR (mg/g), median (IQR)	970 (477, 1845)	936 (477, 1908)
Diabetes (yes), *n* (%)	965 (67.7)	1941 (67.4)
Diabetes duration (years), median (IQR)	14 (8, 21)	14 (7, 21)
CV disease (yes), *n* (%)	475 (33.3)	1135 (39.4)
ACE inhibitor/ARB (yes), *n* (%)	1380 (96.8)	2794 (97.0)
Diuretics (yes), *n* (%)	580 (40.7)	1302 (45.2)
Insulin (yes), *n* (%)*	570 (59.1)	1028 (53.0)

*ACE* angiotensin receptor enzyme, *ARB* angiotensin receptor blocker, *BMI* body mass index, *CV* cardiovascular, *DBP* diastolic blood pressure, *eGFR* estimated glomerular filtration, *SBP* systolic blood pressure, *UACR* urinary albumin-creatinine ratio

*In those with diabetes

### Effects of Dapagliflozin by Age Group

**Figure 1:**
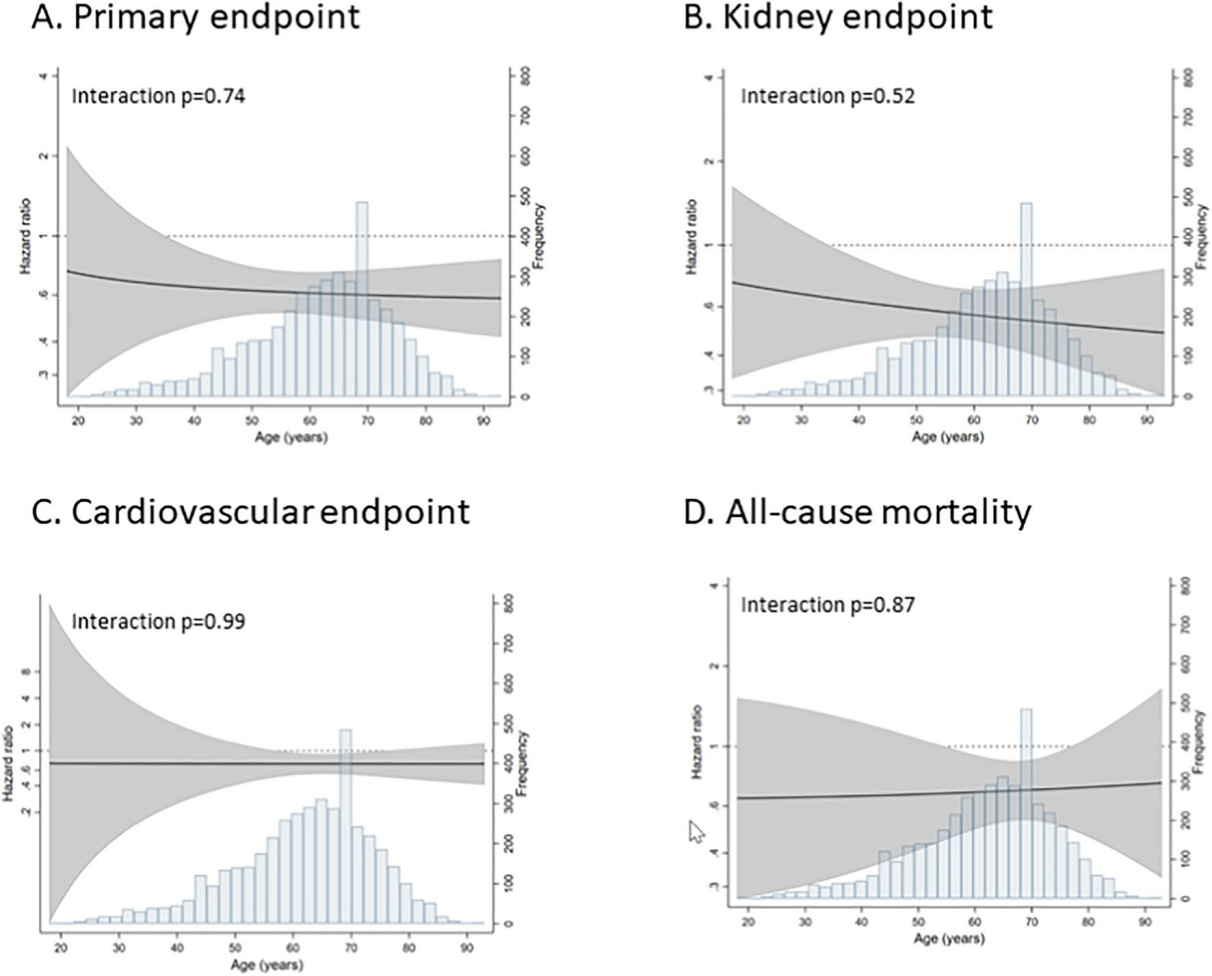
**A**–**D** Interaction between treatment and age for primary endpoint and secondary endpoints.

Effects of dapagliflozin on the primary composite and secondary endpoints by age group are shown in Supplementary Table S1. The absolute risk of the primary composite endpoint was highest in the age group < 50 years (10.7 and 6.5 per 100 patient-years in the placebo and dapagliflozin groups, respectively). Dapagliflozin consistently reduced the risk of the primary composite outcome across age groups; there was no effect modification by age when analyzed as a categorical or continuous variable (Fig. 1A–D). The absolute risks of the kidney composite endpoint were highest in patients < 50 years of age (10.7 and 6.2 per 100 patient-years in the placebo and dapagliflozin groups, respectively) and lowest in patients ≥ 80 years (3.0 and 1.2 per 100 patient-years in the placebo and dapagliflozin groups, respectively). As expected, absolute risks of the cardiovascular composite endpoint and all-cause mortality were higher among older participants. The consistent effects of dapagliflozin across the range of age were evident in patients with and without diabetes.

**Figure 2:**
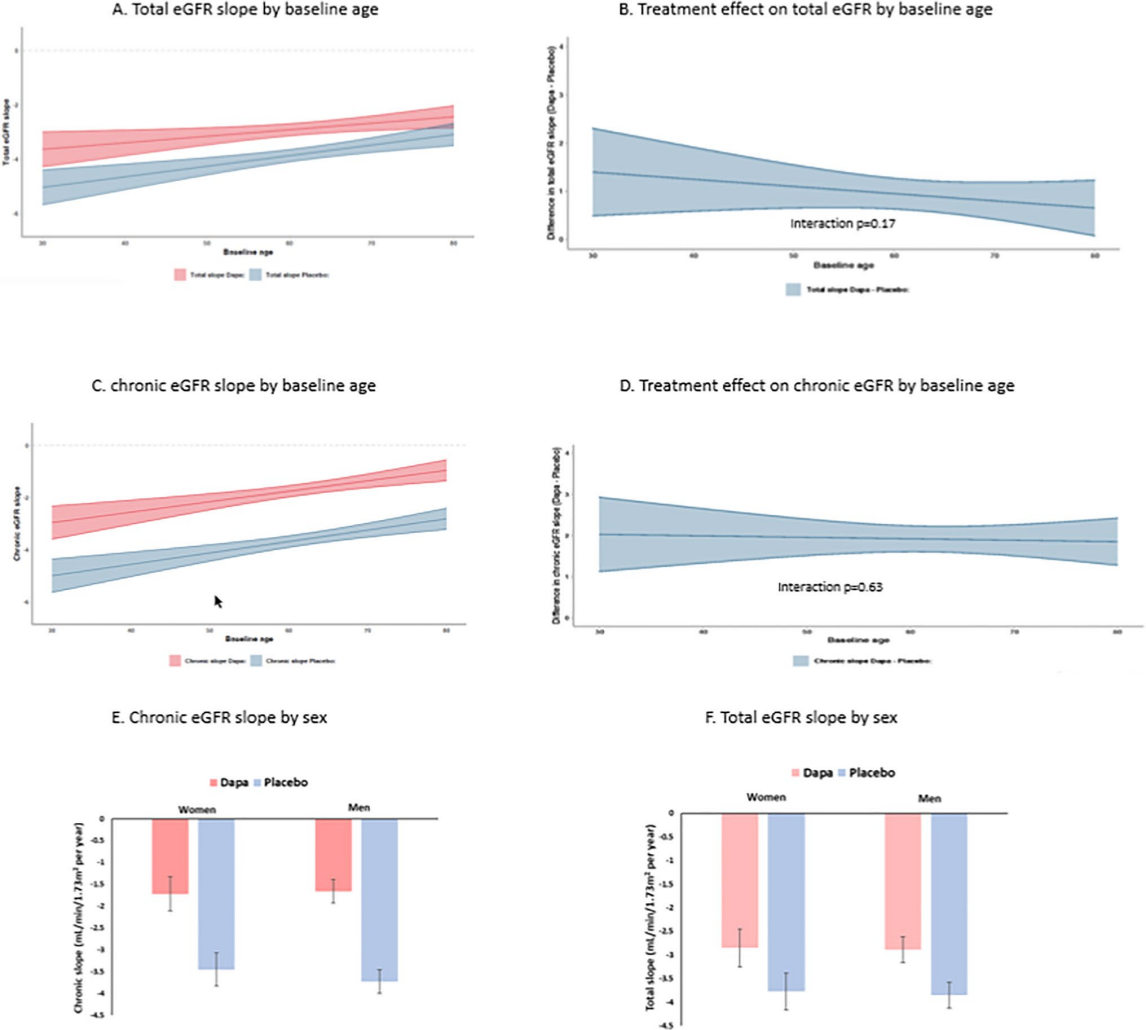
**A**–**F** Effects of dapagliflozin on total or chronic eGFR slope by age and sex.

Age did not modify the effect of dapagliflozin on total or chronic eGFR slope (Fig. 2B, D).

### Effects of Dapagliflozin by Sex

The risk of the primary composite outcome was similar between women and men (Supplementary Table S2). Dapagliflozin reduced the risk of the primary composite endpoint in women (HR 0.65, 95% CI 0.48, 0.88) and men (HR 0.57, 95% CI 0.46, 0.72) with no effect modification by sex (interaction *p* = 0.50). The effect of dapagliflozin on the composite kidney endpoint was similar in women (HR 0.61, 95% CI 0.43, 0.87) and men (HR 0.52, 95% CI 0.40, 0.67; interaction *p* = 0.45), as was the effect on all-cause mortality (women: HR 0.66, 95% CI 0.42, 1.05; men: HR 0.70, 95% CI 0.52, 0.96; interaction *p* = 0.82). In contrast, the effect of dapagliflozin on the cardiovascular composite endpoint appeared to be more pronounced in women (HR 0.47; 95% CI 0.29, 0.75) compared with men (HR 0.86; 95% 0.63, 1.17; interaction *p* = 0.036). Diabetes status did not modify the sex treatment interaction for the primary composite endpoint (interaction *p* = 0.49) or for the three secondary endpoints (interaction *p* = 0.12, 0.59, and 0.90).

Sex did not modify the effect of dapagliflozin on total or chronic eGFR slope (Fig. 2E, F).

### Safety of Dapagliflozin by Age Group and Sex

Serious adverse events and adverse events of special interest by age group and sex are shown in Tables 3 and 4, respectively. Serious adverse events were more frequent in older patients, as expected, and numerically less frequent in dapagliflozin-treated patients relative to those treated with placebo (633 versus 729). There was a numerical increase in the number of fractures in the dapagliflozin group (85 versus 69); fracture events were not adjudicated.

**Table 3:** Safety by Age Categories

Outcome, *n* (%)	Dapagliflozin (*n* = 2148)	Placebo (*n* = 2149)	Odds ratio (95% CI)
Age < 50 years (*n* = 671)	334	337	
Age 50–59 years (*n* = 934)	473	461	
Age 60–69 years (*n* = 1499)	748	751	
Age 70–79 years (*n* = 997)	499	498	
Age ≥ 80 years (*n* = 197)	95	102	
Discontinuation due to adverse event			
Age < 50 years (*n* = 671)	19 (5.7)	15 (4.4)	1.38 (0.68, 2.81)
Age 50–59 years (*n* = 934)	21 (4.4)	26 (5.6)	0.78 (0.43, 1.40)
Age 60–69 years (*n* = 1499)	41 (5.5)	35 (4.7)	1.20 (0.75, 1.91)
Age 70–79 years (*n* = 997)	29 (5.8)	37 (7.4)	0.75 (0.45, 1.25)
Age ≥ 80 years (*n* = 197)	8 (8.4)	10 (9.8)	0.92 (0.34, 2.48)
Any serious adverse event*			
Age < 50 years (*n* = 671)	62 (18.6)	81 (24.0)	0.74 (0.51, 1.08)
Age 50–59 years (*n* = 934)	115 (24.3)	149 (32.3)	0.67 (0.50, 0.90)
Age 60–69 years (*n* = 1499)	237 (31.7)	249 (33.2)	0.94 (0.75, 1.16)
Age 70–79 years (*n* = 997)	179 (35.9)	199 (40.0)	0.84 (0.65, 1.08)
Age ≥ 80 years (*n* = 197)	40 (42.1)	51 (50.0)	0.75 (0.42, 1.32)
Adverse events of interest			
Amputation^†^			
Age < 50 years (*n* = 671)	3 (0.9)	2 (0.6)	1.40 (0.23, 8.63)
Age 50–59 years (*n* = 934)	12 (2.5)	17 (3.7)	0.68 (0.32, 1.44)
Age 60–69 years (*n* = 1499)	13 (1.7)	12 (1.6)	1.09 (0.49, 2.40)
Age 70–79 years (*n* = 997)	7 (1.4)	6 (1.2)	1.17 (0.39, 3.49)
Age ≥ 80 years (*n* = 197)	0	2 (2.0)	NC
Any definite or probable diabetic ketoacidosis			
Age < 50 years (*n* = 671)	0	1 (0.3)	NC
Age 50–59 years (*n* = 934)	0	1 (0.2)	NC
Age 60–69 years (*n* = 1499)	0	0	NC
Age 70–79 years (*n* = 997)	0	0	NC
Age ≥ 80 years (*n* = 197)	0	0	NC
Fracture^‡^			
Age < 50 years (*n* = 671)	5 (1.5)	1 (0.3)	5.29 (0.64,43.86)
Age 50–59 years (*n* = 934)	21 (4.4)	9 (1.9)	2.34 (1.06, 5.17)
Age 60–69 years (*n* = 1499)	35 (4.7)	25 (3.3)	1.43 (0.84, 2.41)
Age 70–79 years (*n* = 997)	17 (3.4)	29 (5.8)	0.56 (0.30, 1.03)
Age ≥ 80 years (*n* = 197)	7 (7.4)	5 (4.9)	1.55 (0.48, 5.07)
Renal-related adverse event^‡^			
Age < 50 years (*n* = 671)	13 (3.9)	28 (8.3)	0.46 (0.23, 0.91)
Age 50–59 years (*n* = 934)	28 (5.9)	49 (10.6)	0.53 (0.32, 0.86)
Age 60–69 years (*n* = 1499)	54 (7.2)	50 (6.7)	1.10 (0.74, 1.64)
Age 70–79 years (*n* = 997)	49 (9.8)	53 (10.6)	0.90 (0.60, 1.36)
Age ≥ 80 years (*n* = 197)	11 (11.6)	8 (7.8)	1.71 (0.66, 4.39)
Major hypoglycamia^§^			
Age < 50 years (*n* = 671)	0	0	NC
Age 50–59 years (*n* = 934)	0	4 (0.9)	NC
Age 60–69 years (*n* = 1499)	7 (0.9)	16 (2.1)	0.43 (0.18, 1.06)
Age 70–79 years (*n* = 997)	7 (1.4)	8 (1.6)	0.85 (0.31, 2.38)
Age ≥ 80 years (*n* = 197)	0	0	NC
Volume depletion^‡^			
Age < 50 (*n* = 671)	20 (6.0)	8 (2.4)	2.58 (1.12, 5.91)
Age 50–59 (*n* = 934)	26 (5.5)	12 (2.6)	2.18 (1.08, 4.38)
Age 60–69 years (*n* = 1499)	37 (4.9)	38 (5.1)	0.98 (0.61, 1.56)
Age 70–79 years (*n* = 997)	35 (7.0)	27 (5.4)	1.32 (0.78, 2.21)
Age ≥ 80 years (*n* = 197)	9 (9.5)	5 (4.9)	2.20 (0.68, 7.09)

^*^Includes death

^†^Surgical or spontaneous/non-surgical amputation, excluding amputation due to trauma

^‡^Based on pre-defined list of preferred terms

^§^Adverse event with the following criteria confirmed by the investigator: (i) symptoms of severe impairment in consciousness or behavior, (ii) need of external assistance, (iii) intervention to treat hypoglycemia, (iv) prompt recovery of acute symptoms following the intervention

*NC* not calculable

**Table 4:** Safety by Sex

Outcome, n (%) Female (*n* = 1425) Male (*n* = 2873)	Dapagliflozin (*n* = 2148)	Placebo (*n* = 2149)	Odds ratio (95% CI)
Discontinuation due to adverse event			
Female (*n* = 1425)	46 (6.5)	42 (5.9)	1.12 (0.73, 1.73)
Male (*n* = 2873)	72 (5.0)	81 (5.6)	0.89 (0.64, 1.24)
Any serious adverse event*			
Female (*n* = 1425)	204 (28.8)	241 (33.7)	0.80 (0.64, 1.00)
Male (*n* = 2873)	429 (29.8)	488 (34.0)	0.82 (0.70, 0.96)
Adverse events of interest			
Amputation^†^			
Female (*n* = 1425)	10 (1.4)	9 (1.3)	1.11 (0.45, 2.77)
Male (*n* = 2873)	25 (1.7)	30 (2.1)	0.82 (0.48, 1.41)
Any definite or probable diabetic ketoacidosis			
Female (*n* = 1425)	0	0	NC
Male (*n* = 2873)	0	2 (0.1)	NC
Fracture^‡^			
Female (*n* = 1425)	37 (5.2)	34 (4.7)	1.11 (0.69, 1.79)
Male (*n* = 2873)	48 (3.3)	35 (2.4)	1.38 (0.89, 2.15)
Renal-related adverse event^‡^			
Female (*n* = 1425)	52 (7.3)	63 (8.8)	0.82 (0.56, 1.21)
Male (*n* = 2873)	103 (7.1)	125 (8.7)	0.81 (0.62, 1.07)
Major hypoglycemia^§^			
Female (*n* = 1425)	4 (0.6)	10 (1.4)	0.40 (0.13, 1.30)
Male (*n* = 2873)	10 (0.7)	18 (1.3)	0.55 (0.25, 1.19)
Volume depletion^‡^			
Female (*n* = 1425)	38 (5.4)	28 (3.9)	1.39 (0.84, 2.29)
Male (*n* = 2873)	89 (6.2)	62 (4.3)	1.46 (1.05, 2.04)

^*^Includes death

^†^Surgical or spontaneous/non-surgical amputation, excluding amputation due to trauma

^‡^Based on pre-defined list of preferred terms

^§^Adverse event with the following criteria confirmed by the investigator: (i) symptoms of severe impairment in consciousness or behavior, (ii) need of external assistance, (iii) intervention to treat hypoglycemia, (iv) prompt recovery of acute symptoms following the intervention

*NC* not calculable

## DISCUSSION

In this pre-specified analysis of data from the DAPA-CKD trial, we demonstrated that dapagliflozin reduced the risks of CKD progression, hospitalized heart failure or cardiovascular death, and all-cause mortality in women and men, and across the spectrum of age, including septuagenarians and octogenarians, who comprised more than 25% of the trial participants. Serious adverse events were more frequent in older patients, but rates were similar in patients treated with dapagliflozin and placebo.

Dozens of studies have demonstrated lower usage of clinically indicated cardiovascular medications in older relative to younger patients and in women relative to men.^9–11, 15–20^ Fewer studies have explored differential usage of medications for CKD—in part owing to the fact that until recently, only ACE inhibitors or ARBs have been shown to be of material benefit in this population; however, sex and age disparities in patients with CKD have been described. In the Chronic Renal Insufficiency Cohort Study, a multicenter, prospective, observational cohort study of adults with mild to moderate CKD (eGFR 20–70 mL/min/1.73 m^2^), women were less likely to be prescribed ACE inhibitors or ARBs at baseline compared with men (63.7 vs 72.9%).^10^ In a cross-sectional analysis of patients with eGFR < 60 mL/min/1.73 m^2^ from the Chronic Kidney Disease Outcomes and Practice Patterns Study, older age and female sex were both associated with lower rates of RAAS inhibitor prescriptions.^19^ In a large cohort of veterans with non-dialysis-dependent CKD, patients treated with ACE inhibitors or ARBs were younger than the untreated patients.^20^

Similarly, studies of real-world utilization of SGLT2 inhibitors consistently found that SGLT2 inhibitors are less frequently prescribed to older versus younger patients and also to women compared to men.^21–26^ In a large, national sample of over 170,000 US veterans with type 2 diabetes and comorbid CKD or cardiovascular disease, Gregg et al. found that the odds of SGLT2 inhibitor prescription were approximately 4% lower for each additional year of age and were 40% lower in women compared to men.^21^ In two separate observational studies of commercially insured patients with type 2 diabetes in the USA, older age and female sex were associated with lower rates of SGLT2 inhibitor use in multivariable analyses.^22, 23^ Rikin et al. found that in a cohort of patients with type 2 diabetes and albuminuria followed in primary care, women experienced a wider care gap with respect to SGLT2 inhibitor prescriptions compared with men.^26^ In a qualitative research study of primary care physicians, age was cited as a significant factor influencing physicians’ decision-making regarding whether to prescribed SGLT2 inhibitors, with a tendency to avoid prescribing SGLT2 inhibitors to older adults out of concern for polypharmacy and adverse effects.^27^

Our study findings are congruent with earlier studies regarding the broad cardiovascular and kidney benefits across age groups and sex in patients with type 2 diabetes and heart failure. The original cardiovascular outcome trials for SGLT2 inhibitors in patients with type 2 diabetes seemed to be broadly beneficial based on subgroup analyses by age and sex.^2, 28–30^ Subsequent analyses have found that the efficacy and safety of dapagliflozin spans age and sex groups in patients with type 2 diabetes,^31, 32^ heart failure with preserved ejection fraction,^33, 34^ and heart failure with reduced ejection fraction.^34–36^

Yi et al. recently published data from the CREDENCE trial, examining the effects of canagliflozin in patients with type 2 diabetes, eGFR 30−90 mL/min/1.73 m^2^ and UACR > 300−5000 mg/g across three categories of age (< 60, 60–< 70, and ≥ 70 years) and by sex.^37^ The authors showed no heterogeneity by age or sex in the primary composite endpoint and several secondary endpoints, which were similar but not identical to those from DAPA-CKD. Our findings confirm those of Yi et al., and extend them to a population that included patients with non-diabetic CKD and patients with lower levels of eGFR, including a sizeable fraction of patients with CKD stage G4.^38^ Our study provides support for extending SGLT2 inhibitor use broadly to patients with advanced CKD irrespective of age or sex. It is noteworthy that both CREDENCE and DAPA-CKD observed similar patterns in racial composition by age and sex, with younger participants more likely to be Asian, older participants more likely to be White, and with more Black women than men enrolled.

This study has several strengths. Data were derived from a randomized trial and major kidney and cardiovascular events were adjudicated by an independent panel. Trial participants were diverse by age and sex as well as country of origin and primary cause of kidney disease. The majority of participants were on guideline-recommended therapies at baseline; nearly all participants were treated with RAAS inhibitors and other agents proven to reduce rates of cardiovascular disease. Effects of dapagliflozin were consistent across the spectrum of age and by sex whether considering risks of discrete events (which are generally observed among “rapid progressors”) as well as eGFR slope. There was a broad inclusion criterion vis-à-vis age, with a sizeable proportion of patients < 50 and > 80 years. The study also has several limitations. First, while we can definitively state that there was no evidence of heterogeneity of the dapagliflozin effect according to age and sex (with the possible exception of the secondary cardiovascular composite endpoint by sex), the power to detect interactions was limited, and we may have missed subtle but clinically meaningful accentuation or attenuation of effects that might be evident in larger patient samples. Second, the DAPA-CKD trial was stopped early following a recommendation from the Independent Data Monitoring Committee. As a result, the trial accrued fewer than 75% of its anticipated number of events; thus, the precision of our estimated treatment effects within each age and sex subgroup was diminished. Third, we did not collect eGFR data after the completion of the trial, which might have increased the observed difference in eGFR slope among age and sex groups, assuming that a fraction of the initial decline observed in dapagliflozin-treated patients could be reversible. Finally, although we enrolled patients with a broad range of underlying kidney diseases, we cannot generalize our results to patients with type 1 diabetes, polycystic kidney disease, and other conditions requiring active immunosuppressive therapy who were excluded from participation.

In summary, dapagliflozin reduced the risks of mortality, cardiovascular events, and CKD progression in women and men, and among patients across the spectrum of age. Perhaps most importantly, older patients, including in septuagenarians and octogenarians who comprised more than 25% of the participants enrolled in DAPA-CKD, experienced clinically meaningful relative and absolute benefits related to treatment. Ageism and/or therapeutic nihilism should not discourage the use of dapagliflozin in older women and men who are likely to experience considerable benefit.

### Supplementary Information

ESM 1 (DOCX 22 kb)
