# Supplementary material for: Effects of Dapagliflozin in Chronic Kidney Disease Across the Spectrum of Age and by Sex
Source: J Gen Intern Med. 2023 Dec 14;39(6):921–30. doi: 10.1007/s11606-023-08397-9 (PMC11074069; doi:10.1007/s11606-023-08397-9)
Supplement: Supplementary file 1 — (DOCX 22 kb) [file 11606_2023_8397_MOESM1_ESM.docx]

**Supplementary appendices**

**Supplementary Table S1. Effects of dapagliflozin compared with placebo on clinical events by age categories**

|  | **Dapagliflozin**  **(n=2152)** | | **Placebo**  **(n=2152)** | | **Absolute Risk Difference (95% CI)** | **Interaction P** | **Hazard Ratio**  **(95% CI);  p value** | **Interaction P** |
| --- | --- | --- | --- | --- | --- | --- | --- | --- |
|  | Age <65 years (n=1170)  Age 65-75 years (n=740)  Age >75 years (n=242) | | Age <65 years (n=1141)  Age 65-75 years (n=765)  Age >75 years (n=246) | |  |  |  |  |
|  | No. (%) | Participants with Event/100 Patient-Yr | No. (%) | Participants  with Event/100 Patient-Yr |  |  |  |  |
| **Primary composite outcome and individual components** | | | | | | | |  |
| *eGFR decline ≥50%, end-stage kidney disease, or kidney or cardiovascular death* | | | | | | | | 0.42 |
| Age <50 years (n=671) | 39 (11.7) | 6.5 | 64 (19.0) | 10.7 | 7.3 (1.9, 12.7) | 0.431 | 0.66 (0.45, 0.99)  p=0.044 |  |
| Age 50-59 years (n=935) | 45 (9.5) | 4.7 | 76 (16.5) | 8.4 | 7.0 (2.7, 11.3) |  | 0.52 (0.36, 0.75)  p<0.001 |  |
| Age 60-69 years (n=1501) | 66 (8.8) | 4.3 | 88 (11.7) | 5.8 | 2.9 (-0.2, 5.9) |  | 0.76 (0.55, 1.05)  p=0.097 |  |
| Age 70-79 years (n=999) | 38 (7.6) | 3.7 | 67 (13.4) | 7.0 | 5.8 (2.0, 9.6) |  | 0.50 (0.33, 0.74)  p=0.001 |  |
| Age ≥80 years (n=198) | 9 (9.4) | 5.2 | 17 (16.7) | 8.6 | 7.3 (-2.0, 16.6) |  | 0.72 (0.32, 1.64)  p=0.440 |  |
| **Secondary outcomes** | | | | | | | |  |
| *eGFR decline ≥50%, end-stage kidney disease or kidney death* | | | | | | | | 0.29 |
| Age <50 years (n=671) | 37 (11.1) | 6.2 | 64 (19.0) | 10.7 | 7.9 (2.5, 13.3) | 0.173 | 0.63 (0.42, 0.95)  p=0.026 |  |
| Age 50-59 years (n=935) | 34 (7.2) | 3.5 | 64 (13.9) | 7.1 | 6.7 (2.8, 10.6) |  | 0.46 (0.30, 0.70)  p<0.001 |  |
| Age 60-69 years (n=1501) | 46 (6.1) | 3.0 | 62 (8.2) | 4.1 | 2.1 (-0.5, 4.7) |  | 0.74 (0.50, 1.08)  p=0.120 |  |
| Age 70-79 years (n=999) | 23 (4.6) | 2.3 | 47 (9.4) | 4.9 | 4.8 (1.7, 8.0) |  | 0.41 (0.25, 0.68)  p<0.001 |  |
| Age ≥80 years (n=198) | 2 (2.1) | 1.2 | 6 (5.9) | 3.0 | 3.8 (-1.6, 9.2) |  | 0.48 (0.09, 2.46)  p=0.378 |  |
| *Cardiovascular death or hospitalization for heart failure* | | | | | | | | 0.92 |
| Age <50 years (n=671) | 4 (1.2) | 0.6 | 3 (0.9) | 0.4 | -0.3 (-1.8, 1.2) | 0.097 | 1.39 (0.31, 6.22)  p=0.666 |  |
| Age 50-59 years (n=935) | 17 (3.6) | 1.6 | 24 (5.2) | 2.4 | 1.6 (-1.0, 4.2) |  | 0.66 (0.35, 1.22)  p=0.183 |  |
| Age 60-69 years (n=1501) | 41 (5.5) | 2.5 | 57 (7.6) | 3.6 | 2.1 (-0.4, 4.6) |  | 0.72 (0.48, 1.07)  p=0.103 |  |
| Age 70-79 years (n=999) | 29 (5.8) | 2.7 | 38 (7.6) | 3.6 | 1.8 (-1.3, 4.9) |  | 0.72 (0.45, 1.17)  p=0.189 |  |
| Age ≥80 years (n=198) | 9 (9.4) | 4.6 | 16 (15.7) | 8.0 | 6.3 (-2.8, 15.5) |  | 0.61 (0.26, 1.42)  p=0.252 |  |
| *All-cause mortality* | | | | | | | | 0.71 |
| Age <50 years (n=671) | 3 (0.9) | 0.4 | 7 (2.1) | 1.0 | 1.2 (-0.6, 3.0) | 0.620 | 0.48 (0.12, 1.87)  p=0.290 |  |
| Age 50-59 years (n=935) | 20 (4.2) | 1.9 | 23 (5.0) | 2.3 | 0.8 (-1.9, 3.5) |  | 0.84 (0.46, 1.54)  p=0.580 |  |
| Age 60-69 years (n=1501) | 36 (4.8) | 2.2 | 55 (7.3) | 3.3 | 2.5 (0.1, 4.9) |  | 0.66 (0.43, 1.00)  p=0.053 |  |
| Age 70-79 years (n=999) | 27 (5.4) | 2.4 | 44 (8.8) | 4.1 | 3.4 (0.2, 6.6) |  | 0.58 (0.36, 0.94)  p=0.028 |  |
| Age ≥80 years (n=198) | 15 (15.6) | 7.5 | 17 (16.7) | 8.1 | 1.0 (-9.2, 11.3) |  | 0.94 (0.46, 1.92)  p=0.872 |  |

PE: age*DM*trt=0.24

SE1: age*DM*trt=0.41

SE2: age*DM*trt=0.36

SE3: age*DM*trt=0.68

**Supplementary Table S2. Effects of dapagliflozin compared with placebo on clinical events by sex**

|  | **Dapagliflozin**  **(n=2152)** | | **Placebo**  **(n=2152)** | | | **Absolute Risk Difference (95% CI)** | **Interaction P** | **Hazard Ratio**  **(95% CI);  p value** | **Interaction P** |
| --- | --- | --- | --- | --- | --- | --- | --- | --- | --- |
|  | Female (n=709)  Male (n=1443) | | Female (n=716)  Male (n=1436) | | |  |  |  |  |
|  | No. (%) | Participants with Event/100 Patient-Yr | | No. (%) | Participants with Event/100 Patient-Yr |  |  |  |  |
| **Primary composite outcome and individual components** | | | | | | | | |  |
| *eGFR decline ≥50%, end-stage kidney disease, or kidney or cardiovascular death* | | | | | | | | | 0.495 |
| Female (n=1425) | 71 (10.0) | 5.0 | | 103 (14.4) | 7.5 | 4.4 (1.0, 7.8) | 0.489 | 0.65 (0.48, 0.88)  p=0.006 |  |
| Male  (n=2879) | 126 (8.7) | 4.4 | | 209 (14.5) | 7.4 | 5.8 (3.5, 8.2) |  | 0.57 (0.46, 0.72)  p<0.001 |  |
| **Secondary outcomes** | | | | | | | | |  |
| *eGFR decline ≥50%, end-stage kidney disease or kidney death* | | | | | | | | | 0.445 |
| Female (n=1425) | 52 (7.3) | 3.6 | | 81 (11.3) | 5.9 | 4.0 (1.0, 7.0) | 0.567 | 0.61 (0.43, 0.87)  p=0.006 |  |
| Male  (n=2879) | 90 (6.2) | 3.2 | | 162 (11.3) | 5.8 | 5.0 (3.0, 7.1) |  | 0.52 (0.40, 0.67)  p<0.001 |  |
| *Cardiovascular death or hospitalization for heart failure* | | | | | | | | | 0.036 |
| Female (n=1425) | 25 (3.5) | 1.6 | | 50 (7.0) | 3.3 | 3.5 (1.1, 5.8) | 0.083 | 0.47 (0.29, 0.75)  p=0.002 |  |
| Male (n=2879) | 75 (5.2) | 2.5 | | 88 (6.1) | 2.9 | 0.9 (-0.8, 2.6) |  | 0.86 (0.63, 1.17)  p=0.328 |  |
| *All-cause death* | | | | | | | | | 0.821 |
| Female (n=1425) | 31 (4.4) | 1.9 | | 45 (6.3) | 2.9 | 1.9 (-0.4, 4.2) | 0.855 | 0.66 (0.42, 1.05)  p=0.081 |  |
| Male  (n=2879) | 70 (4.8) | 2.3 | | 101 (7.0) | 3.3 | 2.2 (0.5, 3.9) |  | 0.70 (0.52, 0.96)  p=0.024 |  |

PE: sex*DM*trt=0.491

SE1: sex*DM*trt=0.123

SE2: sex*DM*trt =0.594

SE3: sex*DM*trt=0.899
